# Supplementary material for: Low-cost otolaryngology simulation models for early-stage trainees: a scoping review
Source: BMC Med Educ. 2024 May 1;24:483. doi: 10.1186/s12909-024-05466-3 (PMC11062898; doi:10.1186/s12909-024-05466-3)
Supplement: Supplementary file 1 — Supplementary Material 1 [file 12909_2024_5466_MOESM1_ESM.docx]

**Searches ran on 2/21/2023**

**OVID**

Database:  **Embase**1974 to 2023 February 20

| **#** | **Searches** | **Results** |
| --- | --- | --- |
| 1 | exp simulation training/ or simulation.ti,ab. or simulator.ti,ab. or simulated.ti,ab. | 466275 |
| 2 | exp otorhinolaryngology/ or otolaryngology.ti,ab. or otorhinolaryngology.ti,ab. or exp ear nose throat surgery/ or ENT.ti,ab. or exp ear/ or exp nose/ or exp throat/ or exp pharynx/ or exp trachea/ or ear.ti,ab. or nose.ti,ab. or throat.ti,ab. or pharynx.ti,ab. or trachea.ti,ab. or cricothyroidostomy.ti,ab. or cricothyrotomy.ti,ab. or exp epistaxis/ or epistaxis.ti,ab. or exp tympanoplasty/ or tympanoplasty.ti,ab. or tympanostomy.ti,ab. or exp tracheostomy/ or tracheostomy.ti,ab. or exp tracheotomy/ or tracheotomy.ti,ab. | 592639 |
| 3 | exp "cost control"/ or low fidelity.ti,ab. or low-fidelity.ti,ab. or cost.ti,ab. or low cost.ti,ab. or low-cost.ti,ab. or price.ti,ab. or inexpensive.ti,ab. or expensive.ti,ab. or cheap.ti,ab. or scarce.ti,ab. or cost-effective.ti,ab. or cost effective.ti,ab. or exp "cost effectiveness analysis"/ or low resource.ti,ab. or low-resource.ti,ab. or exp manikin/ or manikin.ti,ab. or mannequin.ti,ab. | 1104993 |
| 4 | 1 and 2 and 3 | **755** |

**OVID**

Database: **Ovid MEDLINE(R) ALL** 1946 to February 20, 2023

| 1 | exp simulation training/ or simulation.ti,ab. or simulator.ti,ab. or simulated.ti,ab. | 414755 |
| --- | --- | --- |
| 2 | exp Otolaryngology/ or exp Otorhinolaryngologic Surgical Procedures/ or ENT.ti,ab. or exp Ear/ or exp Nose/ or exp Pharynx/ or exp Trachea/ or ear.ti,ab. or nose.ti,ab. or throat.ti,ab. or pharynx.ti,ab. or trachea.ti,ab. or otolaryngology.ti,ab. or otorhinolaryngology.ti,ab. or cricothyroidostomy.ti,ab. or cricothyrotomy.ti,ab. or exp Epistaxis/ or epistaxis.ti,ab. or exp Tympanoplasty/ or tympanoplasty.ti,ab. or exp Middle Ear Ventilation/ or tympanostomy.ti,ab. or exp Tracheostomy/ or tracheostomy.ti,ab. or exp Tracheotomy/ or tracheotomy.ti,ab. | 516525 |
| 3 | exp Cost Savings/ or low fidelity.ti,ab. or low-fidelity.ti,ab. or cost.ti,ab. or low cost.ti,ab. or low-cost.ti,ab. or price.ti,ab. or inexpensive.ti,ab. or expensive.ti,ab. or cheap.ti,ab. or scarce.ti,ab. or cost-effective.ti,ab. or cost effective.ti,ab. or exp Cost-Effectiveness Analysis/ or low resource.ti,ab. or low-resource.ti,ab. or exp Manikins/ or mannequin.ti,ab. or manikin.ti,ab. | 763393 |
| 4 | 1 and 2 and 3 | **626** |

**OVID**

Databases:  **EBM Reviews - Cochrane Central Register of Controlled Trials**January 2023, **Cochrane Database of Systematic Reviews** 2005 to February 15, 2023

| **#** | **Searches** | **Results** |
| --- | --- | --- |
| 1 | exp simulation training/ or simulation.ti,ab. or simulator.ti,ab. or simulated.ti,ab. | 17336 |
| 2 | exp Otolaryngology/ or exp Otorhinolaryngologic Surgical Procedures/ or ENT.ti,ab. or exp Ear/ or exp Nose/ or exp Pharynx/ or exp Trachea/ or ear.ti,ab. or nose/ti,ab. or throat.ti,ab. or pharynx.ti,ab. or trachea.ti,ab. or otolaryngology.ti,ab. or otorhinolaryngology.ti,ab. or cricothyroidostomy.ti,ab. or cricothyrotomy.ti,ab. or exp Epistaxis/ or epistaxis.ti,ab. or exp Tympanoplasty/ or tympanoplasty.ti,ab. or exp Middle Ear Ventilation/ or tympanostomy.ti,ab. or exp Tracheostomy/ or tracheostomy.ti,ab. or exp Tracheotomy/ or tracheotomy.ti,ab. | 27272 |
| 3 | exp Cost Savings/ or low fidelity.ti,ab. or low-fidelity.ti,ab. or cost.ti,ab. or low cost.ti,ab. or low-cost.ti,ab. or price.ti,ab. or inexpensive.ti,ab. or expensive.ti,ab. or cheap.ti,ab. or scarce.ti,ab. or cost-effective.ti,ab. or cost effective.ti,ab. or exp Cost-Effectiveness Analysis/ or low resource.ti,ab. or low-resource.ti,ab. or exp Manikins/ or mannequin.ti,ab. or manikin.ti,ab. | 75284 |
| 4 | 1 and 2 and 3  EBM Reviews - Cochrane Central Register of Controlled Trials <January 2023> 187 | **187** |

**PubMed**

| **#** | **Searches** | **Results** |
| --- | --- | --- |
| 1 | ("Simulation Training"[Mesh] OR simulation [tiab] OR simulator [tiab] OR simulated [tiab]) AND  ("Otolaryngology"[Mesh] OR "Otorhinolaryngologic Surgical Procedures"[Mesh] OR ENT [tiab] OR "Ear"[Mesh] OR "Nose"[Mesh] OR "Pharynx"[Mesh] OR "Trachea"[Mesh] OR ear [tiab] OR nose [tiab] OR throat [tiab] OR pharynx [tiab] OR trachea [tiab] OR otolaryngology [tiab] OR otorhinolaryngology [tiab] OR cricothyroidostomy [tiab] OR cricothyrotomy [tiab] OR "Epistaxis"[Mesh] OR epistaxis [tiab] OR "Tympanoplasty"[Mesh] OR tympanoplasty [tiab] OR "Middle Ear Ventilation" OR tympanostomy [tiab] OR "Tracheostomy"[Mesh] OR tracheostomy [tiab] OR "Tracheotomy"[Mesh] OR tracheotomy [tiab]) AND  ("Cost Savings"[Mesh] OR low fidelity [tiab] OR low-fidelity [tiab] OR cost [tiab] OR low cost [tiab] OR low-cost [tiab] OR price [tiab] OR inexpensive [tiab] OR expensive [tiab] OR cheap [tiab] OR scarce [tiab] OR "Cost-Effectiveness Analysis"[Mesh] OR cost-effective [tiab] OR cost effective [tiab] OR low resource [tiab] OR low-resource [tiab] OR "Manikins"[Mesh] OR mannequin [tiab] OR manikin [tiab]) | **657** |

**Scopus**

| **#** | **Searches** | **Results** |
| --- | --- | --- |
| 1 | TITLE-ABS ( ( simulation OR simulator OR simulated ) AND ( otorhinolaryngology OR otolaryngology OR ent OR ear OR nose OR throat OR pharynx OR trachea OR cricothyroidostomy OR cricothyrotomy OR epistaxis OR tympanoplasty OR tympanostomy OR tracheostomy OR tracheotomy ) AND ( {low fidelity} OR {low-fidelity} OR cost OR {low cost} OR {low-cost} OR price OR inexpensive OR expensive OR cheap OR scarce OR {cost-effective} OR {cost effective} OR {low resource} OR {low-resource} OR manikin OR mannequin ) ) | **942** |

**Web of Science**

| **#** | **Searches** | **Results** |
| --- | --- | --- |
| 1 | TS=(simulation OR simulator OR simulated) AND  (otorhinolaryngology OR otolaryngology OR ENT OR ear OR nose OR throat OR pharynx OR trachea OR cricothyroidostomy OR cricothyrotomy OR epistaxis OR tympanoplasty OR tympanostomy OR tracheostomy OR tracheotomy) AND  ("low fidelity" OR "low-fidelity" OR cost OR "low cost" OR "low-cost" OR price OR inexpensive OR expensive OR cheap OR scarce OR "cost-effective" OR "cost effective" OR "low resource" OR "low-resource" OR manikin OR mannequin) | **806** |

**3973 total references**

| MEDLINE | 626 |
| --- | --- |
| Embase | 755 |
| PubMed | 657 |
| CCTR & CDSR | 187 |
| Scopus | 942 |
| Web of Science | 806 |

**3354 in EndNote after removing conference abstracts**

| MEDLINE | 626 |
| --- | --- |
| Embase | 482 |
| PubMed | 657 |
| CCTR & CDSR | 166 |
| Scopus | 664 |
| Web of Science | 759 |
